# Hot-Stage Microscopy for Determination of API Particles in a Formulated Tablet

**DOI:** 10.1155/2014/832452

**Published:** 2014-07-21

**Authors:** Michal Šimek, Veronika Grünwaldová, Bohumil Kratochvíl

**Affiliations:** ^1^Department of Solid State Chemistry, Institute of Chemical Technology Prague, Technická 5, 166 28 Prague, Czech Republic; ^2^Zentiva k.s., U Kabelovny 130, 102 37 Prague, Czech Republic

## Abstract

Although methods exist to readily determine the particle size distribution (PSD) of an active pharmaceutical ingredient (API) before its formulation into a final product, the primary challenge is to develop a method to determine the PSD of APIs in a finished tablet. To address the limitations of existing PSD methods, we used hot-stage microscopy to observe tablet disintegration during temperature change and, thus, reveal the API particles in a tablet. Both mechanical and liquid disintegration were evaluated after we had identified optimum milling time for mechanical disintegration and optimum volume of water for liquid disintegration. In each case, hot-stage micrographs, taken before and after the API melting point, were compared with image analysis software to obtain the PSDs. Then, the PSDs of the APIs from the disintegrated tablets were compared with the PSDs of raw APIs. Good agreement was obtained, thereby confirming the robustness of our methodology. The availability of such a method equips pharmaceutical scientists with an in vitro assessment method that will more reliably determine the PSD of active substances in finished tablets.

## 1. Introduction

Particle size distribution is one of the most important physical parameters of the starting materials as well as the finished products of pharmaceutical solid dosage forms [1]. On the one hand, particle size distribution of a pharmaceutical solid governs its bulk properties such as flowability, which determines the processability of the starting and intermediate materials and finished products during the manufacturing process. On the other hand, particle size distribution (PSD) of either the excipients or the active pharmaceutical ingredients (APIs) has a profound effect on drug dissolution, bioavailability, stability, and content uniformity, thereby determining the safety, efficacy, and quality of the finished products. Therefore, the analysis and control of particle size distribution of both the excipients and APIs are often an essential part of pharmaceutical product development [2–4].

Tablet formulations typically contain, in addition to an API, sugars and a number of insoluble excipients, which may include microcrystalline cellulose, magnesium stearate, calcium phosphate anhydrous, pigments, and other ingredients. However, excipients often exhibit a broad PSD and a substantial number of excipients may occur in the same size range as the drug substance. This complicates determination of the drug substance. In addition, the majority of APIs and excipients are white powders with rarely predictable particle shape and size. Morphology is usually the only useful clue on how to distinguish API particles in almost all cases of white substances. This leads to inclusion of excipient particles into assessment of an API PSD [5]. In relation to limitations of existing PSD methods, we investigated a methodology for PSD determination based on hot-stage microscopy.

Hot-stage microscopy has been already used for the characterization of pharmaceutical substances. For example, Vitez et al. used hot-stage microscopy for observing polymorphic phase transformations of an API [6]. It is frequently used as a complementary technique to DSC during compatibility analyses [7]. Similar usage of this technique can be easily found in the literature. It is obvious that hot-stage microscopy is becoming a routine analytical tool for system observation over temperature [8].

Spectral techniques such as FTIR or Raman mapping are sometimes used for the approximation of particle size of drug substance in a tablet [9]. These techniques require a high quality cut of tablet. Methods of the cut preparation are described in our previous puplication [10]. A result map provides the spatial distributions of the various components within a sample by different colors. However, this does not preclude API particles agglomerate formation and the detection of agglomerates as single particles. It should be noted that images often cannot differentiate between a large particle and agglomerated particles [11]. Thus, a chemical distribution within the image is often described in terms of the “domain” size rather than the particle size [12, 13]. Comparison of the results of spectral mapping and hot-stage microscopy could be useful for discovering API agglomerates in a drug product.

## 2. Materials

### 2.1. Raw Materials

We used tadalafil and meloxicam as model APIs. Tadalafil is a phosphodiesterase-5 inhibitor used for a treatment of the erectile dysfunction [14–17]. Meloxicam is a nonsteroidal anti-inflammatory drug with an analgesic and fever reducer effect [18]. Common excipients were used with APIs to create an experimental mixture and model tablets. The list of compounds used and their melting points are shown in Table 1, as well as the composition of the mixture and tablets which are made of them.

### 2.2. Mixture 1

Mixture 1 consists of tadalafil (50%) and Methocel (50%). Mixture 1 was used for the demonstration of hot-stage microscopy as a useful technique in early stage of our work.

### 2.3. TFL Tbl 1

Tablets TFL Tbl 1 were prepared by direct compression of tadalafil (10%), calcium carbonate (39%), lactose monohydrate (46.2%), silicon dioxide (2.7%), and magnesium stearate (2.1%).

### 2.4. ME Tbl 1

Tablets ME Tbl 1 were prepared by direct compression of meloxicam (9.9%), lactose monohydrate (15.3%), Avicel PH102 (50.3%), silicon dioxide (1.6%), crospovidone (14.4%), and magnesium stearate (9.6%).

### 2.5. ME Tbl 2

Tablets ME Tbl 2 were prepared by compression of granules. Povidone was used as a granulation binder. Granules were made of meloxicam (9.6%), lactose monohydrate (14.9%), Avicel PH102 (48.8%), silicon dioxide (1.5%), crospovidone (13.9%), magnesium stearate (9.3%), and povidone (3%).

## 3. Methods

A tablet must first be disintegrated before analysis as hotstage microscopy was used for observation of solid powder mixture over change in temperature.

### 3.1. Mechanical Disintegration of Tablets

The mechanical method of powder preparation from a tablet is shortly described in the publication from Koradia et al. A small portion of a tablet core is pressed lightly between two glass slides. We consider this procedure suitable only for softer tablets [8]. Conversely, mechanical crushing of a tablet may cause breaking of a tablet as well as individual particles. Because of this, it was necessary to assess the dependency of particles size reduction on milling time.

### 3.2. Liquid Disintegration of Tablets

This method of disintegration is based on the dissolution of soluble components followed by the filtration and drying of insoluble components. Selected dissolution liquid must not dissolve the API. Elimination of soluble components causes increase of API content in the prepared powder. This is advantageous for API's identification by a microscope, especially in the case of tablets with a low amount of API [19]. The dependency of the API particle size change on a volume of used disintegration liquid was one of our experimental aims.

### 3.3. Analytical Methods

#### 3.3.1. Size Analysis

The evaluations of the particle or domain size distribution of APIs were performed with image analysis (NIS Elements 4.11 software, LIM—Laboratory imaging spol. s.r.o., Za drahou 171/17, Prague, Czech Republic) of hot-stage micrographs as well as spectral images. Approximately 600 particles were evaluated in every analysis. Particle size distributions are often reported by parameters based upon the maximum particle size for a given percentage (10, 50, and 90%) of sample. For this reason, PSDs were compared by percentile *d*-values which are known as the lower decil—*d*(0.1), median—*d*(0.5), and upper decil—*d*(0.9).

#### 3.3.2. Hot-Stage Micrographs

Light microscope Nikon eclipse Ni with LTS420 temperature controlled stage (Nikon spol. s.r.o., K Radotínu 14, Prague, Czech Republic) was used for experiments with 4x and 10x lens. Heating rate was 10°C per minute.

#### 3.3.3. Spectral Images

FTIR mapping was carried out using Nicolet iN10 MX Infrared Imaging Microscope (Thermo Fisher Scientific-Thermo Scientific Inc., Vienna, Austria) with OmnicSpecta software (Thermo Scientific Inc., Vienna, Austria). Full performance leads to resolution of 6.25 *μ*m per pixel.

## 4. Results and Discussion

The aim of this paper was to show hot-stage microscopy as a suitable tool for the identification of API particles in a mixture and disintegrated tablet. Important part of this work was focused on finding the best parameters for tablet disintegration by the mechanical and liquid disintegration method. To find out the best parameters, the dependencies of the *d*-values of PSDs were observed. Each experiment, including selection of 600 API particles, was repeated five times and variation obtained.

### 4.1. Identification of API with Hot-Stage Microscopy

**Figure 1:**
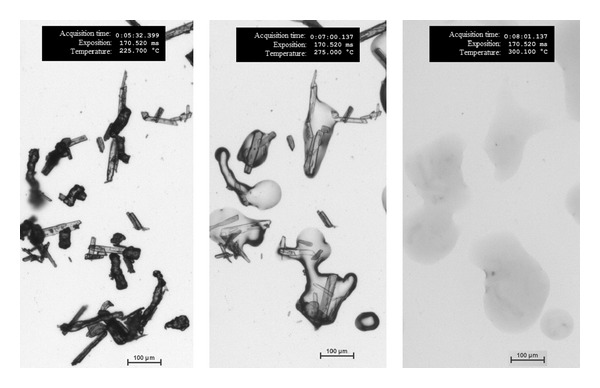
Mixture 1 at 225°C, 275°C, and 300°C temperatures. No change of the system was observed from 25 to 225°C. Methocel melted at 230°C and tadalafil melted at 295°C.

The principle of API identification is shown on the example of mixture 1 (Figure 1). As tadalafil has higher melting point than Methocel, needle-shaped particles of tadalafil melt later. API particles were identified by comparing pictures taken at 275°C and 300°C as the melting point of tadalafil is 295°C. The experiments were repeated until 600 particles of tadalafil were analyzed.

**Figure 2:**
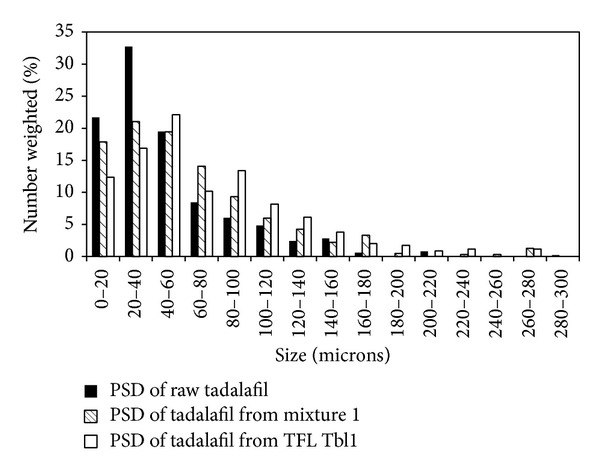
PSDs of raw tadalafil, tadalafil in mixture 1 and in TFL Tbl 1 tablets.

Tablet TFL Tbl 1 was disintegrated to powder by a few drops of water. Prepared powder was filtered and dried at room temperature and used for the analyses as described. Three PSDs of tadalafil (raw, in mixture 1, and in TFL Tbl 1) are compared in Figure 2. The PSDs have similar range and occurrence of particle sizes.

### 4.2. Mechanical Disintegration of Tablets

**Figure 3:**
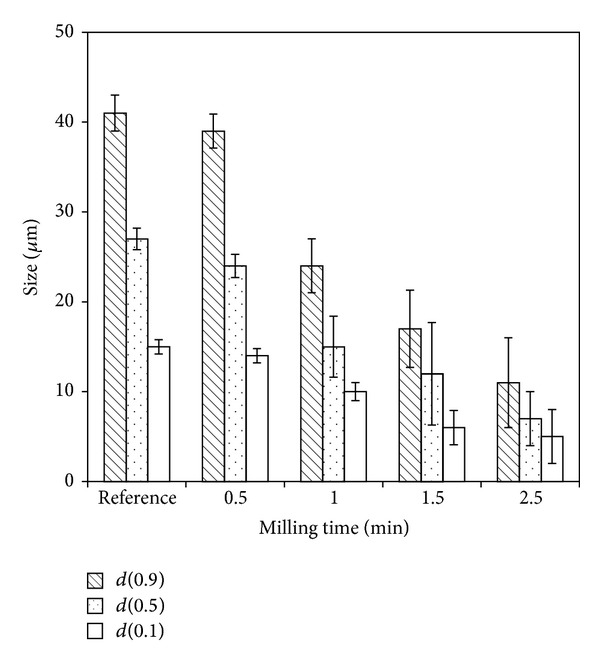
Change of *d*(0.1), *d*(0.5), and *d*(0.9) of API particle size distributions over milling time. Reference values belong to raw meloxicam.

Tablets ME Tbl 2 were prepared by the compression of granules and were used for experiments. An adverse phenomenon of the mechanical method is gradual destruction of particles over the milling time.  Figure 3 shows the change of *d*-values of meloxicam particle size distributions over milling time. Reference values of raw meloxicam were measured before formulation into tablets. Minimal change (approximately 5 percent) of *d*-values was observed at 0.5 min milling time. The rapid decrease of particle size was observed if the milling time was longer than 0.5 min.

At first, a tablet is broken into small pieces. Then, small pieces are milled into granules. Till some granules are presented, smooth milling does not significantly destroy individual particles. This is the reason why some residual granules were presented in prepared samples and they were discarded during image analyses.

### 4.3. Liquid Disintegration of Tablets

**Figure 4:**
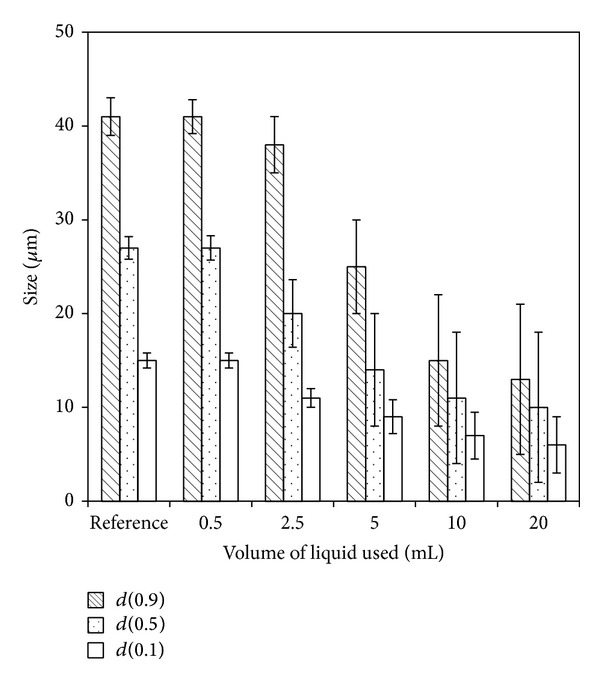
Change of *d*(0.1), *d*(0.5), and *d*(0.9) of meloxicam PSDs over volume of water used. Reference values belong to raw meloxicam.

Because it was found that granulation binders, used in the granulation process, caused hardening of prepared powder, directly compressed tablets ME Tbl 1 were used for these experiments.  Figure 4 shows change of *d*-values of meloxicam PSDs over volume of water used for the disintegration. PSD analyses were carried out using the method described in mechanical disintegration. Almost no change of *d*-values was observed if 0.5 mL of water was used. This volume equals the volume being required to dissolve all sufficiently soluble components of ME Tbl 1 tablet [20]. It can be calculated as a saturated solution of all sufficiently soluble components in a tablet [21]. In our case, more than 0.5 mL of water caused partial dissolution of meloxicam particles.

### 4.4. Comparison of Hot-Stage Microscopy and FTIR Mapping

**Figure 5:**
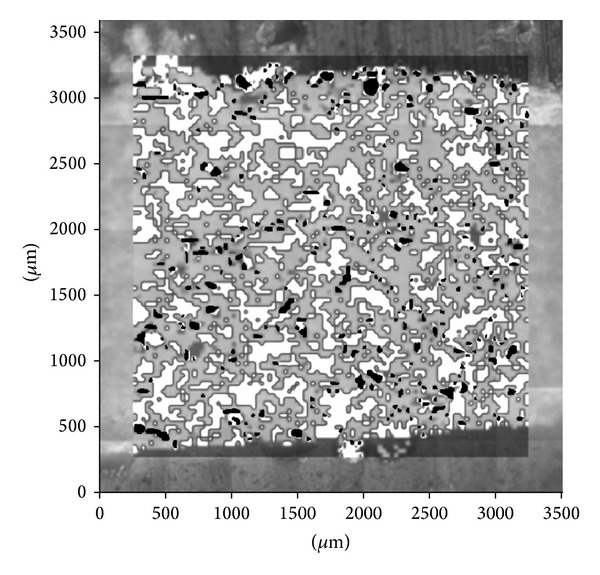
FTIR map of ME Tbl 1 cut. (meloxicam—black, Avicel PH102—dark grey, lactose—light grey, and crospovidone—white).

**Figure 6:**
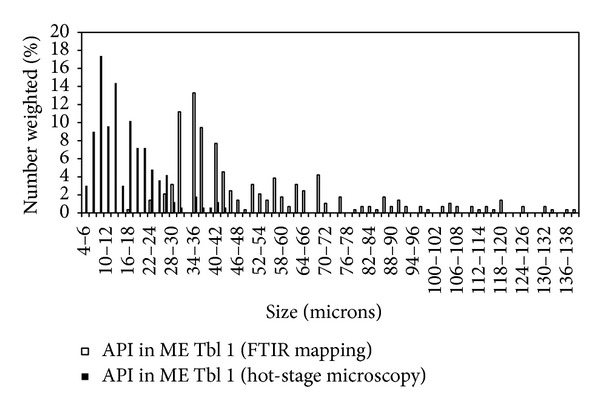
Results of meloxicam PSD in generic drug (ME Tbl 1) obtained by hot-stage microscopy and by FTIR mapping.

The preparation of tablet cut is described in our previous publication [10]. FTIR map of the microtome cut of ME Tbl 1 tablet is shown in Figure 5. FTIR mapping presents “domains” of all components (meloxicam—black, Avicel PH102—dark grey, lactose—light grey, and crospovidone—white). Image analysis results of meloxicam PSD, obtained from FTIR mapping and hot-stage microscopy, are compared in Figure 6. A lot of API “domains” from FTIR map are much bigger than particles analyzed with hot-stage microscopy which provides congruent results with reference PSD of meloxicam. FTIR mapping does not seem to be suitable for an API PSD assessment. On the other hand, comparison of FTIR map with hot-stage microscopy allows the determination of API agglomerates in a tablet.

### 4.5. Application of Hot-Stage Microscopy Analysis (Case Study)

**Figure 7:**
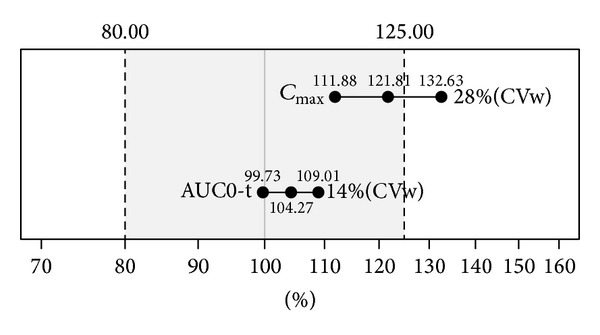
Results of failed bioequivalence study. The maximal concentration in blood (*C*_max⁡_) of generic product is out of the tolerance limit. Area under the curve of pharmacokinetic profile (AUC) complies.

**Figure 8:**
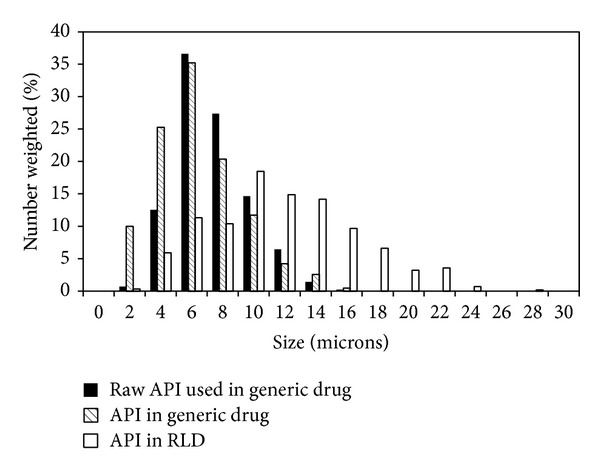
Comparison of PSD of raw API used in generic drug, API in generic tablet, and API in the RLD tablet.

The aim of this part is to demonstrate the application of hot-stage microscopy analysis as a routine analytical method in the pharmaceutical industry. Bioequivalence studies are performed to demonstrate in vivo that two pharmaceutically equivalent products (in the US) or alternative pharmaceutical products (in the EU) are comparable in their rate and extent of absorption [22]. For reaching the bioequivalence, values of the maximal concentration in blood (*C*_max⁡_), the area under the curve of pharmacokinetic profile (AUC), and their deviations, must be situated inside the tolerance limit. Width of the limit (from 80 to 125%) is defined by the regulatory authorities and center point (100%) represents relative values of *C*_max⁡_ and AUC of the reference listed drug (RLD). As shown in Figure 7, the bioequivalence study of generic and reference listed drug (RLD) was not reached. We used hot-stage microscopy to compare API PSD in the RLD and generic tablet and raw API used in the generic tablet (Figure 8). The results showed a difference in PSDs of API used in the RLD and generic tablet and helped to find out why the bioequivalent study had not matched in *C*_max⁡_. Higher *C*_max⁡_ of the generic tablet was truly caused by the smaller size of API particles, as the smaller particles dissolved faster and led to higher *C*_max⁡_. API particles in the RLD are roughly double the size of the particles in the generic tablet. Almost identical PSDs of the raw API and API in the generic product confirm the robustness of the method.

## 5. Conclusion

The experiments demonstrated that hot-stage microscopy and image analysis allow identification of API particles in mixtures or in disintegrated tablets. The methodology is based on different melting points of individual substances. The disintegration of tablet into powder is essential for PSD analysis by hot-stage microscopy. The mechanical disintegration was suitable for both—tablets produced by direct compression and those compressed from granules. However, it is not recommended to completely grind a tablet into powder. If all bigger particles of a tablet were milled, individual API particles were partially broken. The liquid disintegration was suitable only for directly compressed tablets. In case of compressed granules, granulation binders caused hardening of the prepared powder. The API should be insoluble or, at least, minimally soluble in liquid. The qualitative and quantitative composition of a tablet are required for the right calculation of the liquid volume. The volume of the liquid is calculated like the volume of the saturated solution of all soluble excipients in a tablet. It was found that more API particles are partially dissolved when a volume greater than the calculated liquid volume is used. The elution of soluble excipients allows the API content in the prepared powder to be increased. The comparison of results produced by hot-stage microscopy and FTIR mapping is considered a useful way to identify API agglomerates in a tablet. Importantly, hot-stage microscopy can be used as a routine tool in pharmaceutical development for comparing particle size of the RLD and generic product.

## Figures and Tables

**Table 1:** List of used APIs, excipients, their melting points, and composition of mixture and tablets.

	Tadalafil	Meloxicam	Calcium carbonate	Methocel	Lactose monohydrate	Avicel PH102	Colloidal silicon dioxide	Crospovidone	Magnesium stearate	Povidone
Melting point [°C]	286–295	242–250	825	225–230	214–216	260–275	1600	150–180	117–150	150–180
Mixture 1	●			●						
TFL Tbl 1	●		●		●		●		●	
ME Tbl 1		●			●	●	●	●	●	
ME Tbl 2		●			●	●	●	●	●	●
